# Properly Substituted Benzimidazoles as a New Promising Class of Nicotinate Phosphoribosyltransferase (NAPRT) Modulators

**DOI:** 10.3390/ph16020189

**Published:** 2023-01-27

**Authors:** Cecilia Baldassarri, Gianfabio Giorgioni, Alessandro Piergentili, Wilma Quaglia, Stefano Fontana, Valerio Mammoli, Gabriele Minazzato, Elisa Marangoni, Massimiliano Gasparrini, Leonardo Sorci, Nadia Raffaelli, Loredana Cappellacci, Riccardo Petrelli, Fabio Del Bello

**Affiliations:** 1 Medicinal Chemistry Unit, School of Pharmacy, Chemistry Interdisciplinary Project (ChIP), University of Camerino, Via Madonna delle Carceri, 62032 Camerino, Italy; 2 Center for Drug Discovery and Development-DMPK, Aptuit, an Evotec Company, Via A. Fleming 4, 37135 Verona, Italy; 3 Department of Agriculture, Food and Environmental Sciences, Polytechnic University of Marche, Via Brecce Bianche 10, 60131 Ancona, Italy; 4 Division of Bioinformatics and Biochemistry, Department of Materials, Environmental Sciences and Urban Planning, Polytechnic University of Marche, 60131 Ancona, Italy

**Keywords:** nicotinate phosphoribosyltransferase, substituted benzimidazoles, NAD biosynthesis, NAPRT inhibitors, NAPRT activators, pharmacokinetics

## Abstract

The prevention of nicotinamide adenine dinucleotide (NAD) biosynthesis is considered an attractive therapeutic approach against cancer, considering that tumor cells are characterized by an increased need for NAD to fuel their reprogrammed metabolism. On the other hand, the decline of NAD is a hallmark of some pathological conditions, including neurodegeneration and metabolic diseases, and boosting NAD biosynthesis has proven to be of therapeutic relevance. Therefore, targeting the enzymes nicotinamide phosphoribosyltransferase (NAMPT) and nicotinate phosphoribosyltransferase (NAPRT), which regulate NAD biosynthesis from nicotinamide (NAM) and nicotinic acid (NA), respectively, is considered a promising strategy to modulate intracellular NAD pool. While potent NAMPT inhibitors and activators have been developed, the search for NAPRT modulators is still in its infancy. In this work, we report on the identification of a new class of NAPRT modulators bearing the 1,2-dimethylbenzimidazole scaffold properly substituted in position 5. In particular, compounds **24**, **31**, and **32** emerged as the first NAPRT activators reported so far, while **18** behaved as a noncompetitive inhibitor toward NA (*K*_i_ = 338 µM) and a mixed inhibitor toward phosphoribosyl pyrophosphate (PRPP) (*K*_i_ = 134 µM). From in vitro pharmacokinetic studies, compound **18** showed an overall good ADME profile. To rationalize the obtained results, docking studies were performed on the NAPRT structure. Moreover, a preliminary pharmacophore model was built to shed light on the shift from inhibitors to activators.

## 1. Introduction

**Figure 1:**
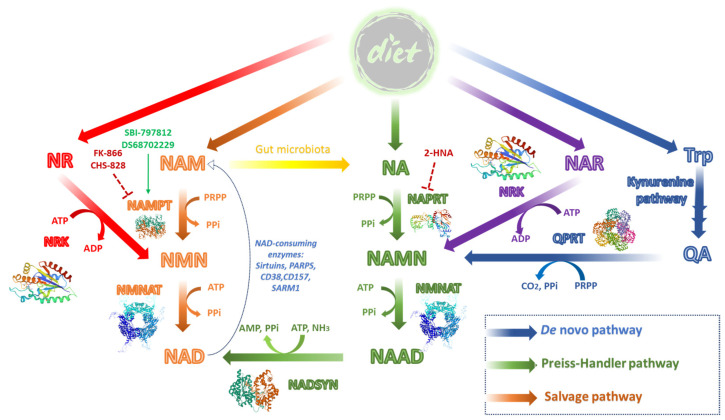
NAD biosynthesis in mammalian cells. NR, nicotinamide riboside; NRK, nicotinamide riboside kinase; NAM, nicotinamide; NAMPT, nicotinamide phosphoribosyltransferase; NMN, nicotinamide mononucleotide; NMNAT, nicotinamide mononucleotide adenylyltransferase; NAD, nicotinamide adenine dinucleotide; NA, nicotinic acid; NAPRT, nicotinate phosphoribosyltransferase; NAMN, nicotinic acid mononucleotide; NAAD, nicotinic acid adenine dinucleotide; NADSYN, nicotinamide adenine dinucleotide synthetase; NAR, nicotinic acid riboside; Trp, tryptophan; QA, quinolinic acid; QPRT, quinolinate phosphoribosyltransferase; PRPP, phosphoribosyl pyrophosphate; PPi, inorganic pyrophosphate. Known inhibitors (in red) or activators (in green) of NAD metabolism are highlighted in the figure.

The long-known universal coenzyme nicotinamide adenine dinucleotide (NAD) plays an important role in energetic metabolism both as a cofactor in redox reactions and as a substrate for NAD-consuming enzymes that regulate critical cellular processes (e.g., inflammatory response, metabolic adaptation, differentiation, and signal transduction) [1,2,3]. Since NAD is cleaved by the catalytic activity of NAD-consuming enzymes, it needs to be continuously replenished. Its biosynthesis is ensured by multiple biosynthetic pathways that start from different precursors, including tryptophan and the three forms of vitamin B3, i.e., nicotinic acid (NA), nicotinamide (NAM), and nicotinamide riboside (NR). As shown in Figure 1, the three main pathways for NAD biosynthesis are the de novo pathway, the Preiss–Handler pathway, and the salvage pathway. Furthermore, the ribosylated precursors NR and NAR represent additional NAD precursors. The various biosynthetic pathways occur in different combinations and with different efficiency depending on the cell type and metabolic status [4].

Cancer cells, which are marked by a heightened need for NAD to fuel their reprogrammed energy metabolism, rely on a sustained NAM salvage pathway through the increased expression and activity of the rate-limiting enzyme nicotinamide phosphoribosyltransferase (NAMPT). In particular, NAMPT is frequently up-regulated in solid and hematological cancers and was the first NAD biosynthetic enzyme for which a clear potential as a drug target was demonstrated [5,6]. Several potent and selective NAMPT inhibitors have been developed (e.g., FK866 and CHS-828), which exhibit strong anti-tumor activity in in vitro and in vivo tumor models by efficiently lowering NAD production in a dose- and time-dependent manner. However, they have failed so far as an anticancer therapy in clinical trials [7,8,9] also because most tumor cells can elude NAMPT blockade by exploiting alternative NAD-producing pathways [10].

**Figure 2:**
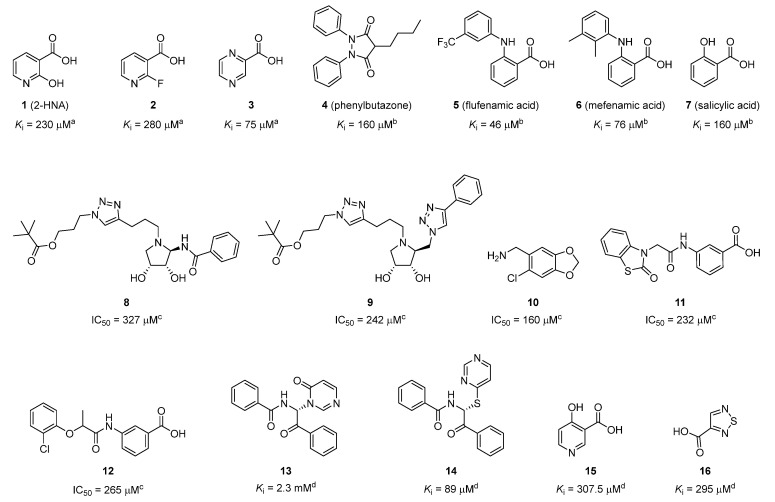
Structures and *K*_i_ or IC_50_ values of the NAPRT inhibitors reported so far. ^a^ Taken from reference [14]. ^b^ Taken from reference [15]. ^c^ Taken from reference [16]. ^d^ Taken from reference [17].

Indeed, overexpression of the enzyme nicotinate phosphoribosyltransferase (NAPRT), which controls the Preiss–Handler pathway, is commonly found in several types of tumors, including ovarian, pancreatic, liver, and colorectal cancers [11]. In these cells, silencing NAPRT expression leads to decreased energy, protein synthesis, and cell size, indicating that NAPRT might also represent a promising anticancer target [10]. Notably, cancer cells expressing high levels of NAPRT are resistant to NAMPT inhibitors. This resistance can be reversed through gene silencing or direct inhibition of NAPRT, suggesting that co-administration of NAPRT and NAMPT inhibitors might represent a successful therapy to decrease NAD levels, leading to cancer cell death [12,13]. To our knowledge, only a few weak NAPRT inhibitors have been identified until now, including 2-hydroxynicotinic acid (2-HNA) and its analogs and nonsteroidal anti-inflammatory drugs such as phenylbutazone, mefenamic acid, flufenamic acid, and salicylic acid (compounds **1–7**, Figure 2) [14,15]. Recently, Franco et al. and Ghanem et al. have identified a new series of NAPRT inhibitors through a structure-based drug design and by in silico drug discovery, respectively (compounds **8–16**, Figure 2). The novel chemical entities showed inhibitory properties against NAPRT comparable to 2-HNA, with *K*i values in the micromolar range [16,17].

Contrary to tumor transformation, metabolic and cardiovascular diseases, as well as neurodegenerative disorders, are associated with a marked decline in NAD levels, and boosting NAD biosynthesis has emerged as a potential therapeutic approach for their treatment [18,19]. Small molecule activators of NAMPT, which are effective in increasing intracellular NAD levels and result in strong neuroprotective efficacy in pre-clinical models, have been developed [20,21]. Regarding NAPRT, activation of the enzyme by substrate supplementation in cultured cells was shown to boost NAD levels and decrease cytotoxicity induced by oxidative stress [22]. However, no enzyme activator has been identified, and therefore the therapeutical potential of the enzyme’s pharmacological activation has not been explored so far.

In this work, by screening more than 200 lead-like molecules available in our labs and characterized by different chemical scaffolds, we have identified the properly substituted 1,2-dimethylbenzimidazole nucleus as a novel chemotype for the preparation of ligands able to modulate NAPRT activity acting as inhibitors or activators that might represent hits for further optimization.

## 2. Results and Discussion

### 2.1. Identification of the Hit Compound

**Figure 3:**
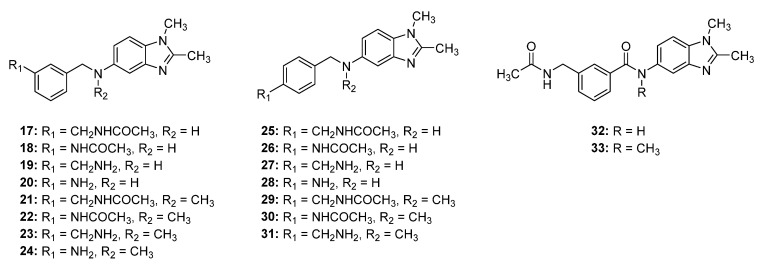
Chemical structure of the new 1,2-dimethylbenzimidazole derivatives **17–33**.

Over 200 small molecules available in our labs were tested on the activity of human recombinant NAPRT as described in Materials and Methods, and this screening led to the identification of a small set of weak inhibitors. Among them, a compound bearing the 1,2-dimethylbenzimidazole scaffold (compound **17**) (Figure 3) was found to exert 30% inhibition when tested at 1 mM concentration, at saturating concentrations of NA and phosphoribosyl pyrophosphate (PRPP) substrates.

Taking **17** as a starting point, a series of structural analogs were designed and prepared (compounds **18–33**, Figure 3), to develop a comprehensive structure–activity relationship (SAR) investigation and to establish the structural determinants for an efficacious NAPRT inhibition. In particular, to evaluate the role of acetamidomethyl substituent in position 3 of the phenyl ring, this group was replaced by the acetamido, aminomethyl, or amino groups (compounds **18–20**). The same substituents were shifted from position 3 to 4 (compounds **25–28**). Moreover, the role of the CH_2_NH bridge was evaluated by its replacement with an amide function (compound **32**). Finally, to probe the importance of the hydrogen bond donor group in the bridge, the secondary amines/amides were methylated (compounds **21–24**, **29–31**, and **33**).

### 2.2. Synthesis of Compounds ***17–33***

**Scheme 1:**
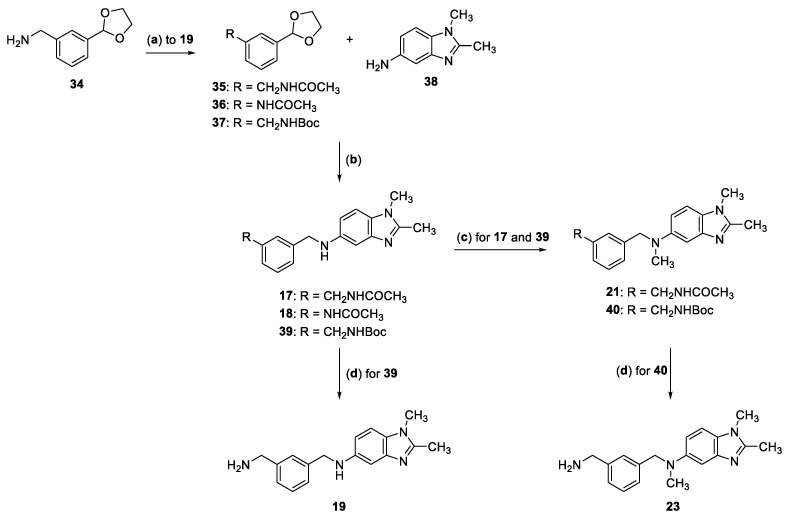
Reagents: (**a**) (CH_3_CO)_2_O, CHCl_3_; (**b**) (i) toluene, Δ; (ii) NaBH_3_CN, ClCH_2_CH_2_Cl; (**c**) HCHO, NaBH_3_CN, ClCH_2_CH_2_Cl; (**d**) 2.5N HCl in MeOH.

Compounds **17–19**, **21**, and **23** were prepared according to the procedure reported in Scheme 1. Amine **34** (Aldrich) was acetylated with acetic anhydride to give amide **35**. Compounds **35**, **36** [23], and **37** [24] were subjected to reductive amination by treatment with amine **38** [25], followed by reduction of the intermediate imine with NaBH_3_CN, affording **17**, **18**, and **39**, respectively. The N-methylation of **17** and **39** with formaldehyde in the presence of NaBH_3_CN yielded the corresponding derivatives **21** and **40**. The cleavage of the Boc protective group of **39** and **40** led to compounds **19** and **23**, respectively.

**Scheme 2:**
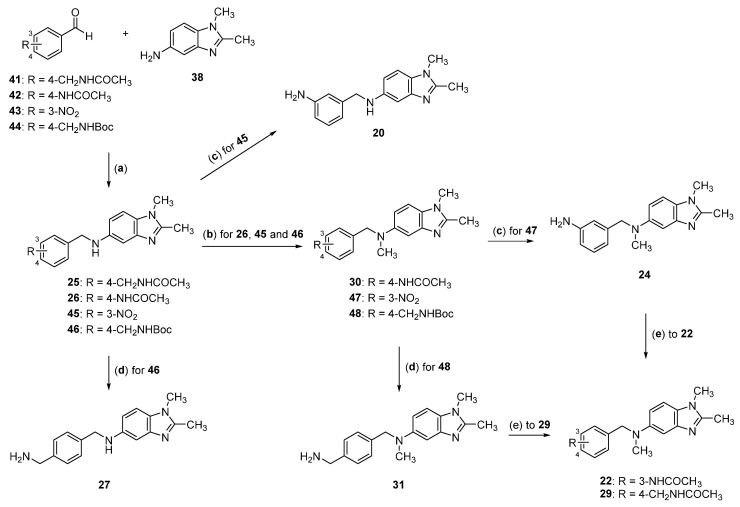
Reagents: (**a**) (i) toluene, Δ; (ii) NaBH_3_CN, ClCH_2_CH_2_Cl; (**b**) HCHO, NaBH_3_CN, ClCH_2_CH_2_Cl; (**c**) H_2_/Raney Nickel, CH_3_OH; (**d**) 2.5N HCl in MeOH; (**e**) (CH_3_CO)_2_O, CHCl_3_.

Compounds **20**, **22**, **24–27**, and **29–31** were prepared according to the procedure reported in Scheme 2. The reaction of the aldehydes **41** [26], **42** (Aldrich), **43** (Aldrich), and **44** (Aldrich) with amine **38**, followed by treatment with NaBH_3_CN gave **25**, **26**, **45**, and **46**, respectively. The treatment of **26**, **45**, and **46** with formaldehyde in the presence of NaBH_3_CN yielded the corresponding *N*-methyl derivatives **30**, **47**, and **48**. Compounds **20** and **24** were obtained by reduction of the nitro group of **45** and **47**, respectively, with H_2_/Raney Nickel. The Boc protective group of **46** and **48** were cleaved with HCl, yielding compounds **27** and **31**, respectively. Compounds **22** and **29** were obtained by acetylation of **24** and **31** with acetic anhydride.

**Scheme 3:**
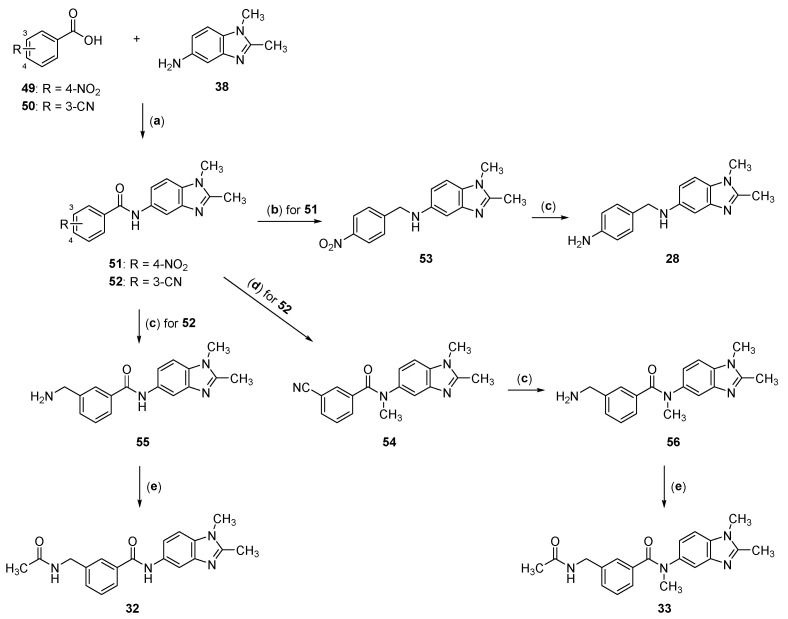
Reagents: (**a**) EDCI·HCl, HOBt, N-methylmorpholine, DMF; (**b**) BH_3_ (CH_3_)_2_S, THF, Δ; (**c**) H_2_/Raney Nickel, CH_3_OH; (**d**) NaH, CH_3_I, DMF; (**e**) (CH_3_CO)_2_O, CHCl_3_.

Compounds **28**, **32**, and **33** were prepared according to the procedure reported in Scheme 3. Amidation of the carboxylic acids **49** and **50** (Aldrich) with amine **38** in the presence of 1-ethyl-3-carbodiimide hydrochloride (EDCI7#xB7;HCl), 1-hydroxybenzotriazole (HOBt) and N-methylmorpholine gave compounds **51** and **52**, respectively. The reduction of the amide group of **51** with borane dimethyl sulfide complex afforded intermediate **53**. The methylation of **52** with methyl iodide in the presence of NaH yielded derivative **54**. Compounds **52**, **53**, and **54** were hydrogenated using Raney Nickel as a catalyst to obtain **55**, **28**, and **56**, respectively. Compounds **32** and **33** were obtained by acetylation of **55** and **56** with acetic anhydride.

### 2.3. Structure–Activity Relationship (SAR) Study

The effect of the synthetized compounds on NAPRT catalytic activity was tested by the HPLC assay described in Materials and Methods. Three compounds (**18**, **25** and **29**) were found to inhibit NAPRT activity, with compound **18** being more effective than the hit compound **17**, whereas, surprisingly, five compounds (**20**, **23**, **24**, **31** and **32**) resulted to be NAPRT activators. The results of the screening are reported in Table 1. From the data analysis, it emerges that the removal of the methylene group between the acetamido function and the phenyl ring of **17**, leading to the lower homolog **18**, causes a slight increase in NAPRT inhibition. The hydrolysis of the amide function of **17** leads to the inactive amine **19**, while, noteworthy, the same modification performed on its lower homolog **18** modulates the profile from NAPRT inhibitor to activator (compound **20**). The methylation of the secondary amine in the bridge decreases the activity of inhibitors (**21** vs. **17** and **22** vs. **18**), while it increases that of the activators (**23** vs. **19** and **24** vs. **20**). 

Different SARs are observed when the substituent is shifted from position 3 to 4 of the phenyl ring. Only compounds **25** and **29**, analogs of **17** and **21**, respectively, behave as very weak inhibitors. In this case, the methylation does not affect the activity, and both compounds show similar % inhibition. Instead, analogously to what is observed with the 3-amine derivatives, the methylation of the 4-amino derivative **27** leads to a potent NAPRT activator (compound **31**). 

Finally, the substitution of the CH_2_NH bridge of **17** with an amide function, yielding **32**, also causes an interesting modulation of the biological profile from NAPRT inhibition to activation. In contrast to the above-reported SARs, the N-methylation in the bridge abolishes the enzyme activation (compound **33**). In general, all the identified inhibitors are characterized by an acetamide terminal, while all the activators, except for the di-amide **32**, bear a free primary amine terminal.

**Figure 4:**
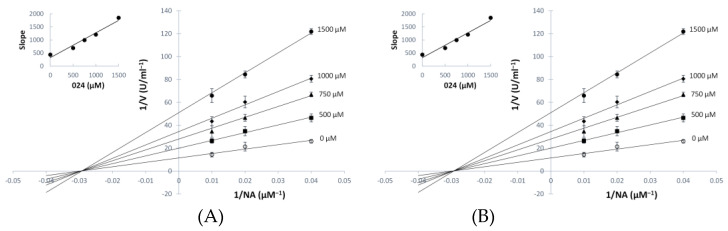
Inhibition of NAPRT by **18**. Double-reciprocal plots and slope replots (in the inserts) of the inhibition exerted by **18** at (**A**) NA concentrations ranging from 25 µM to 100 µM, at 0.4 mM PRPP, and (**B**) PRPP concentrations ranging from 25 µM to 100 µM, at 0.4 mM NA. Each point is the mean of duplicate determinations.

Overall, from this SAR study compounds **24**, **31**, and **32** emerge as the most potent NAPRT activators, while **18** shows the highest inhibition within this series. The effect of compound **18** on NAPRT activity was further confirmed by measuring the *K*_i_ values towards NA and PRPP and defining the inhibition mechanism. As shown in Figure 4, inhibition is noncompetitive towards NA, with a *K*_i_ value of 338 ± 25 µM, and it is mixed towards PRPP, with a *K*_i_ value of 134 ± 13 µM. The *K*_i_ value towards NA is very similar to those reported for the NAPRT inhibitors identified so far [14,15,16]. To our knowledge, this is the first kinetic study that analyzes the effect of a NAPRT inhibitor toward the substrate PRPP. The different types of inhibition towards the two substrates suggest that the inhibitor might in part occupy the PRPP binding site and, in part, stick out of the active site. This is supported by the results from the docking experiments.

### 2.4. In Silico Analysis of Inhibitor and Activator Binding Pocket

**Figure 5:**
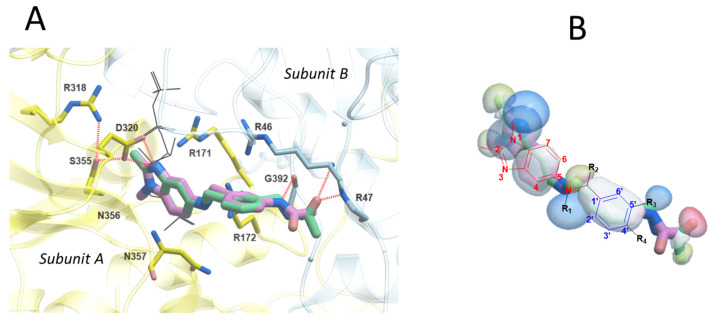
(**A**) Binding modes of inhibitors **17** and **18.** Human dimeric NAPRT protein is represented in ribbon. The two chains are differently labeled and colored (chain A in yellow and chain B in cyan). The two inhibitors (**17**, in green; **18**, in pink) and the residues that interact with the ligands are represented as sticks. H-bonds are indicated as red dotted lines. A modeled PRPP substrate is shown as black wires. (**B**) A simple pharmacophore model of the inhibitory scaffold. The blobs are color-coded: yellow for hydrophobic; white for aromatic; blue for H-bond donor; red for H-bond acceptor.

Following the in vitro study of the benzimidazole-based NAPRT modulators, we performed molecular docking on the NAPRT structure to get insights into the ligand-pocket interactions. The docking of the best inhibitors **17** and **18** show two similar poses that overlap with the PRPP subsite and partly stick out of the active site (Figure 5A). This is consistent with our complete kinetic characterization of inhibitor **18**, which resulted in noncompetitive versus NA and mixed toward PRPP. The benzimidazole ring is ionized at physiological pH and engages Asp320^A^ with a salt bridge and H-bonds. Asp320^A^ is indirectly connected through a tight H-bonding network to Arg318^A^, a residue implicated in catalysis through mutagenesis [27,28]. Intriguingly, Arg318 is also perturbed by two reported inhibitors (**10** and **12** in Figure 2) that share similar chemical moieties with our inhibitory scaffold. The benzimidazole ring system is further held in place by two π-cation interactions with Arg171-2^A^ on one side and by hydrophobic interactions with the main chain of Asn356-7^A^ on the other face. In contrast, the benzene ring is stabilized by a face-to-edge π-cation interaction with Arg46^B^. Outside the active site area, **17** and **18** differently engage the subunit B with H-bonds between the inhibitor’s amide group and the main chains of Gly393^B^ or Arg46^B^ and Arg47^B^, respectively.

### 2.5. Rationale for Modulators’ Activity

We next wanted to rationalize the activity of our series of benzimidazole derivatives. This effort will, hopefully, shed light on the shift from inhibitors to activators and guide their future optimization. To this end, we built a preliminary pharmacophore model with the only two available inhibitors’ binding poses (Figure 5B). This model points to two important H-bond donor and acceptor areas in the phenyl-5′-substituent (R_3_ in Figure 5B). At this level, the activators possess positively charged primary amines or methyl amines that do not fit into the model. Furthermore, activators also tend to have a methyl substituent in position R_1_. Consistently, this last chemical feature, when present, diminishes the activity in the inhibitors series. Indeed, as seen in Figure 5A, such methyl group would be squeezed between Asn357 and Arg172. The docking of activators shows scattered favorable poses outside the active site (not shown). This is in line with their chemical features that do not fit well in the pharmacophore model for inhibition. Future studies that will help us to locate the activator binding site with certainty are required to uncover the activation activity of this chemical series.

### 2.6. In Vitro Pharmacokinetic Studies

The in silico ADME characterization has recently been reported for NAPRT inhibitors **1**, **15** and **16**, belonging to different chemotypes, and the results indicated promising pharmacokinetic features for the studied compounds [17]. With the aim to preliminarily shed light on the pharmacokinetics of this series of compounds, **18** was evaluated for its in vitro ADME profile. Kinetic solubility at pH 7.4, plasma protein binding from human and mouse species determined by using equilibrium dialysis, intrinsic hepatic clearance determined in human and mouse liver microsomes, and permeability studies in MDCKII (Madin–Darby canine kidney cells, a cell strain derived from the distal tubule or collecting duct of the nephron) and MDCKII-MDR1 (MDCK II cells overexpressing the multidrug resistance protein 1, MDR1) cell lines were performed according to previously reported procedures [29,30,31,32]. Both cell lines were originally obtained from American Type Culture Collection (ATCC) and have been cultured and amplified with respect to permeability assessment at the test site.

The results reported in Table 2 reveal that compound **18** shows an overall good in vitro ADME profile. In particular, it is characterized by high kinetic solubility at pH 7.4 (312 μM) and low protein binding in both human and mouse plasma. From the hepatic intrinsic clearance assay, based on the hepatic microsome system, compound **18** proves to be stable in humans while showing a moderate clearance in mouse. It also shows medium apparent permeability in wild-type MDCKII cell lines, but relevant efflux ratios (51.3) when tested in MDCKII-MDR1 cell lines, which are transfected with the MDR1 gene encoding for the efflux protein P-glycoprotein (P-gp). Therefore, this compound proves to be a P-gp substrate.

## 3. Materials and Methods

### 3.1. Chemistry

Materials and general methods for the synthesis and chemical characterization of the final compounds and intermediates are reported in the ‘Supplementary Materials’ and given in references [33,34].

N-(3-(((1,2-dimethyl-1H-benzo[d]imidazol-5-yl)amino)methyl)benzyl)acetamide (**17**)

A mixture of **35** (3.1 mmol) and **38** (3.1 mmol) in toluene (50 mL) was heated to reflux. A Dean–Stark trap was used to collect the water removed by the azeotrope. After 4 h, the mixture was cooled to r.t. and concentrated in a vacuum to remove the toluene. The imine was taken up for reduction without purification (91% yield). It was suspended in dichloroethane (50 mL), cooled to 0 °C and NaBH_3_CN (5.64 mmol) was added in portions. The reaction mixture was stirred overnight at r.t., washed with brine, dried over Na_2_SO_4_, and concentrated to get the crude product, which was purified by flash chromatography eluting with CHCl_3_/MeOH (95:5) to yield an oil (55% yield). The free base was transformed into the hydrochloride salt, which was recrystallized from 2-PrOH to obtain a red solid (m.p. 148–150 °C). ^1^H NMR (DMSO) δ 1.84 (s, 3H, CH_3_CO), 2.38 (s, 3H, CH_3_), 3.57 (s, 3H, NCH_3_), 4.20 (s, 2H, CH_2_N), 4.23 (s, 2H, CH_2_N), 5.83 (br t, 1H, NHCO exchangeable with D_2_O), 6.59–7.28 (m, 7H, ArH) 8.38 (br t, 1H, ArNH exchangeable with D_2_O). ^13^C NMR (DMSO) δ 12.5, 22.9, 30.7, 42.5, 48.6, 100.3, 111.8, 115.6, 124.2, 124.9, 125.1, 126.8, 128.3, 128.7, 137.3, 137.9, 140.9, 150.6, 169.4. ESI/MS *m*/*z* 323 [M + H]^+^. Anal. Calcd. (C_19_H_22_N_4_O.2HCl) C, H, N.

N-(3-(((1,2-dimethyl-1H-benzo[d]imidazol-5-yl)amino)methyl)phenyl)acetamide (**18**)

This compound was prepared starting from **36** and **38** following the procedure described for **17**: an oil was obtained (49% yield). The free base was transformed into the hydrochloride salt, which was recrystallized from 2-PrOH, to obtain a white solid (m.p. 198–200 °C). ^1^H NMR (DMSO) δ 1.94 (s, 3H, CH_3_CO), 2.63 (s, 3H, CH_3_), 3.78 (s, 3H, NCH_3_), 4.27 (s, 2H, CH_2_N), 5.75 (br s, 1H, NHCO exchangeable with D_2_O), 6.61–7.62 (m, 7H, ArH) 10.01 (br s, 1H, ArNH exchangeable with D_2_O). ^13^C NMR (DMSO) δ 12.5, 22.8, 30.7, 48.6, 100.5, 111.7, 115.4, 119.2, 119.7, 122.7, 124.8, 128.6, 128.8, 137.8, 138.3, 141.8, 150.8, 168.9. ESI/MS *m*/*z* 309 [M + H]^+^, 639 [2M + Na]^+^. Anal. Calcd. (C_18_H_20_N_4_O.2HCl) C, H, N.

N-(3-(aminomethyl)benzyl)-1,2-dimethyl-1H-benzo[d]imidazol-5-amine (**19**)

Compound **39** (1.0 mmol) was treated with 2.5N HCl in MeOH (10 mL) and the mixture was stirred at r.t. for 1h. Evaporation of the solvent gave the crude product, which was recrystallized from 2-PrOH, to obtain a red solid (m.p. 250–253 °C). ^1^H NMR (DMSO) δ 2.63 (s, 3H, CH_3_), 3.80 (s, 3H, NCH_3_), 3.92 (br s, 1H, NHAr exchangeable with D_2_O), 4.00 (m, 2H, CH_2_NH_2_), 4.37 (s, 2H, CH_2_NH), 6.60–7.58 (m, 7H, ArH), 8.49 (br s, 2H, NH_2_ exchangeable with D_2_O). ^13^C NMR (DMSO) δ 12.6, 30.5, 46.3, 48.6, 101.1, 111.6, 115.3, 123.9, 124.3, 124.8, 126.7, 128.3, 128.8, 137.8, 141.2, 141.8, 150.8. ESI/MS *m*/*z* 281 [M + H]^+^. Anal. Calcd. (C_17_H_20_N_4_.2HCl) C, H, N.

N-(3-aminobenzyl)-1,2-dimethyl-1H-benzo[d]imidazol-5-amine (**20**) 

Compound **45** (6.72 mmol) was hydrogenated at 40 psi in MeOH for 2 h at r.t. using Raney Nickel (0.02 g) as the catalyst. Following catalyst removal by filtration, the evaporation of the solvent gave a residue which was purified by flash chromatography eluting with CHCl_3_/MeOH (8:2) to yield an oil (91% yield). The free base was transformed into oxalate salt, which was recrystallized from 2-PrOH to obtain a yellow solid (m.p. 165–166 °C). ^1^H NMR (DMSO) δ 2.62 (s, 3H, CH_3_), 3.45 (br s, 3H, NH, NH_2_ exchangeable with D_2_O), 3.78 (s, 3H, NCH_3_), 4.07 (s, 2H, CH_2_N), 6.38–7.59 (m, 7H, ArH). ^13^C NMR (DMSO) δ 12.4, 30.6, 48.4, 100.9, 111.5, 113.5, 113.9, 115.4, 116.8, 124.1, 128.8, 129.3, 137.6, 142.4, 148.2, 150.7. ESI/MS *m*/*z* 267 [M + H]^+^. Anal. Calcd. (C_16_H_18_N_4_.2H_2_C_2_O_4_) C, H, N.

N-(3-(((1,2-dimethyl-1H-benzo[d]imidazol-5-yl)(methyl)amino)methyl)benzyl) acetamide (**21**)

A suspension of **17** (1.6 mmol) in dichloroethane (50 mL) was cooled to 0 °C and formaldehyde (37% solution in water, 0.2 mL, 2.53 mmol) was added dropwise. The solution was stirred for 15 min at 0 °C and then NaBH_3_CN (3.2 mmol) was added in portions. The reaction mixture was stirred overnight at r.t. The crude was diluted with dichloromethane (30 mL), washed with brine, dried over Na_2_SO_4_, and concentrated to get a residue which was purified by flash chromatography eluting with CH_2_Cl_2_/MeOH (95:5) to yield an oil (71% yield). The free base was transformed into oxalate salt, which was recrystallized from 2-PrOH to obtain a white solid (m.p. 130–132 °C). ^1^H NMR (CD_3_OD) δ 1.91 (s, 3H, CH_3_CO), 2.51 (s, 3H, CH_3_), 2.94 (s, 3H, CH_3_NCH_2_), 3.67 (s, 3H, NCH_3_), 4.28 (s, 2H, CH_2_NCO), 4.46 (s, 2H, CH_2_N), 5.83 (br t, 1H, NHCO exchangeable with D_2_O), 6.87–7.23 (m, 7H, ArH). ^13^C NMR (CD_3_OD) δ 11.6, 21.8, 30.4, 39.1, 41.7, 55.4, 98.3, 110.3, 124.3, 125.4, 125.8, 126.4, 128.7, 136.7, 139.4, 140.3, 147.4, 150.1, 163.4, 169.2. ESI/MS *m*/*z* 337 [M + H]^+^. Anal. Calcd. (C_20_H_24_N_4_O.H_2_C_2_O_4_) C, H, N.

N-(3-(((1,2-dimethyl-1H-benzo[d]imidazol-5-yl)(methyl)amino)methyl)phenyl) acetamide (**22**)

Acetic anhydride (3.63 mmol) was dropwise added to a solution of **24** (3.02 mmol) in chloroform (20 mL) under ice cooling. After the reaction was completed, ice water was added, and the mixture was extracted with chloroform (2 × 30 mL). The combined extracts were washed with 2N NaOH and water, dried over Na_2_SO_4_, and concentrated to get a residue which was purified by flash chromatography eluting with CH_2_Cl_2_/MeOH (95:5) to yield an oil (80% yield). The free base was transformed into the hydrochloride salt, which was recrystallized from 2-PrOH to obtain a yellow solid (m.p. 195–197 °C). ^1^H NMR (DMSO) δ 2.01 (s, 3H, CH_3_CO), 2.41 (s, 3H, CH_3_), 2.96 (s, 3H, CH_3_NCH_2_) 3.62 (s, 3H, NCH_3_), 4.41 (s, 2H, CH_2_), 6.78–7.51 (m, 7H, ArH), 9.95 (br s, 1H, NHCO exchangeable with D_2_O). ^13^C NMR (DMSO) δ 12.4, 22.9, 30.7, 39.2, 56.8, 97.7, 111.8, 116.4, 119.6, 120.8, 123.8, 125.7, 128.8, 132.8, 136.6, 138.9, 147.7, 151.4, 169.5. ESI/MS *m*/*z* 323 [M + H]^+^. Anal. Calcd. (C_19_H_22_N_4_O.2HCl) C, H, N.

N-(3-(aminomethyl)benzyl)-N,1,2-trimethyl-1H-benzo[d]imidazol-5-amine (**23**)

This compound was prepared starting from **40** following the procedure described for **19**: an oil was obtained (77% yield). The free base was transformed into the oxalate salt, which was recrystallized from 2-PrOH, to obtain a white solid (m.p. 156–158 °C). ^1^H NMR (CD_3_OD) δ 2.77 (s, 3H, CH_3_), 3.85 (s, 3H, NCH_3_), 4.09 (s, 2H, CH_2_N), 4.68 (s, 2H, CH_2_NH_2_), 6.86–7.58 (m, 7H, ArH), 10.12 (br s, 2H, NH_2_ exchangeable with D_2_O). ^13^C NMR (CD_3_OD) δ 11.6, 30.4, 39.1, 46.7, 55.4, 98.3, 110.3, 124.3, 125.4, 125.8, 126.4, 128.7, 139.4, 140.3, 141.4, 147.4, 150.1, 163.4. ESI/MS *m*/*z* 295 [M + H]^+^. Anal. Calcd. (C_18_H_22_N_4_.2H_2_C_2_O_4_) C, H, N.

N-(3-aminobenzyl)-N,1,2-trimethyl-1H-benzo[d]imidazol-5-amine (**24**)

This compound was prepared starting from **47** following the procedure described for **20**: an oil was obtained (89% yield). The free base was transformed into the oxalate salt, which was recrystallized from 2-PrOH, to obtain a white solid (m.p. 181–182 °C). ^1^H NMR (DMSO) δ 2.41 (s, 3H, CH_3_), 2.95 (s, 3H, CH_3_NCH_2_) 3.60 (s, 3H, NCH_3_), 3.42 (br s, 2H, NH_2_ exchangeable with D_2_O), 4.38 (s, 2H, CH_2_), 6.38–7.22 (m, 7H, ArH). ^13^C NMR (DMSO) δ 12.4, 30.7, 39.2, 56.8, 97.9, 111.8, 113.3, 114.7, 116.4, 118.2, 125.7, 128.8, 129.4, 137.2, 147.7, 148.2, 151.4. ESI/MS *m*/*z* 281 [M + H]^+^. Anal. Calcd. (C_17_H_20_N_4_.H_2_C_2_O_4_) C, H, N.

N-(4-(((1,2-dimethyl-1H-benzo[d]imidazol-5-yl)amino)methyl)benzyl)acetamide (**25**)

This compound was prepared starting from **41** and **38** following the procedure described for **17**: an oil was obtained (49% yield). The free base was transformed into the hydrochloride salt, and recrystallized from i-PrOH to obtain a red solid (m.p. 153–156 °C). ^1^H NMR (CD_3_OD) δ 1.97 (s, 3H, CH_3_CO), 2.42 (s, 3H, CH_3_), 3.58 (s, 3H, NCH_3_), 4.31 (m, 4H, CH_2_), 5.38 (br s, 1H, NHCO), 5.82 (br t, 1H, NH exchangeable with D_2_O), 6.69–7.39 (m, 7H, ArH). ^13^C NMR (CD_3_OD) δ 11.4, 21.3, 27.6, 41.8, 45.8, 99.6, 107.9, 109.3, 126.1, 127.3 (2C), 127.9 (2C) 129.4, 135.4, 136.8, 143.3, 151.9, 168.7. ESI/MS *m*/*z* 323 [M + H]^+^. Anal. Calcd. (C_19_H_22_N_4_O.2HCl) C, H, N.

N-(4-(((1,2-dimethyl-1H-benzo[d]imidazol-5-yl)amino)methyl)phenyl)acetamide (**26**)

This compound was prepared starting from **42** and **38** following the procedure described for **17**: an oil was obtained (49% yield). The free base was transformed into oxalate salt, which was recrystallized from i-PrOH to obtain a white solid (m.p. 168–169 °C). ^1^H NMR (DMSO) δ 2.01 (s, 3H, CH_3_CO), 2.42 (s, 3H, CH_3_), 3.38 (br s, 1H, NH exchangeable with D_2_O), 3.60 (s, 3H, NCH_3_), 4.20 (s, 2H, CH_2_), 6.59–7.50 (m, 7H, ArH), 9.96 (br s, 1H, NHCO exchangeable with D_2_O). ^13^C NMR (DMSO) δ 13.7, 23.7, 29.9, 47.9, 100.1, 109.7, 110.7, 121.3 (2C), 127.7, 128.6 (2C), 130.7, 135.6, 138.5, 143.3, 151.2, 168.9. ESI/MS *m*/*z* 309 [M + H]^+^, 331 [M + Na]^+^. Anal. Calcd. (C_18_H_20_N_4_O.2H_2_C_2_O_4_) C, H, N.

N-(4-(aminomethyl)benzyl)-1,2-dimethyl-1H-benzo[d]imidazol-5-amine (**27**)

This compound was prepared starting from **46** following the procedure described for **19**: an oil was obtained (77% yield). The free base was transformed into the oxalate salt, which was recrystallized from 2-PrOH, to obtain a yellow solid (m.p. 208–210 °C). ^1^H NMR (DMSO) δ 2.61 (s, 3H, CH_3_), 3.79 (s, 3H, NCH_3_), 3.99 (m, 3H, CH_2_NH_2_ and NHAr), 4.38 (s, 2H, CH_2_NH), 6.55–7.63 (m, 7H, ArH), 8.55 (br s, 2H, NH_2_ exchangeable with D_2_O). ^13^C NMR (DMSO) δ 13.7, 29.9, 45.9, 47.9, 100.1, 109.7, 110.7, 127.7, 128.2 (2C), 128.9 (2C), 130.7, 137.9, 141.7, 143.3, 151.2. ESI/MS *m*/*z* 281 [M + H]^+^. Anal. Calcd. (C_17_H_20_N_4_.2H_2_C_2_O_4_) C, H, N.

N-(4-aminobenzyl)-1,2-dimethyl-1H-benzo[d]imidazol-5-amine (**28**).

This compound was prepared starting from **53** following the procedure described for **20**. A red solid was obtained (74% yield; m.p. 171–172 °C). ^1^H NMR (DMSO) δ 2.56 (s, 3H, CH_3_), 3.82 (s, 3H, NCH_3_), 4.15 (s, 2H, CH_2_), 4.67 (br s, 3H, NH, NH_2_ exchangeable with D_2_O), 6.53–7.45 (m, 7H, ArH). ^13^C NMR (DMSO) δ 13.7, 29.9, 47.9, 100.1, 109.7, 110.7, 113.8 (2C), 127.7, 128.7 (2C), 130.7, 130.9, 143.3, 147.7, 151.2. ESI/MS *m*/*z* 267 [M + H]^+^. Anal. Calcd. (C_16_H_18_N_4_) C, H, N.

N-(4-(((1,2-dimethyl-1H-benzo[d]imidazol-5-yl)(methyl)amino)methyl)benzyl) acetamide (**29**)

This compound was prepared starting from **31** following the procedure described for **22**: an oil was obtained (74% yield). The free base was transformed into the hydrochloride salt, which was recrystallized from 2-PrOH, to obtain a red solid (m.p. 147–150 °C). ^1^H NMR (CD_3_OD) δ 1.97 (s, 3H, CH_3_CO), 2.58 (s, 3H, CH_3_), 2.98 (s, 3H, CH_3_NCH_2_), 3.77 (s, 3H, NCH_3_), 4.33 (s, 2H, CH_2_NCO), 4.55 (s, 2H, CH_2_N), 5.82 (br t, 1H, NHCO exchangeable with D_2_O), 6.83–7.37 (m, 7H, ArH). ^13^C NMR (CD_3_OD) δ 11.4, 21.3, 27.6, 40.7, 41.8, 56.7, 100.4, 106.8, 109.4, 125.8, 126.9 (2C), 127.4 (2C), 129.6, 135.7, 136.4, 147.8, 151.9, 169.2. ESI/MS *m*/*z* 337 [M + H]^+^. Anal. Calcd. (C_20_H_24_N_4_O.2HCl) C, H, N.

N-(4-(((1,2-dimethyl-1H-benzo[d]imidazol-5-yl)(methyl)amino)methyl)phenyl) acetamide (**30**)

This compound was prepared starting from **26** following the procedure described for **21**: an oil was obtained (48% yield). The free base was transformed into the hydrochloride salt, which was recrystallized from 2-PrOH, to obtain a white solid (m.p. 163–164 °C). ^1^H NMR (DMSO) δ 2.01 (s, 3H, CH_3_CO), 2.42 (s, 3H, CH_3_), 2.90 (s, 3H, CH_3_NCH_2_), 3.61 (s, 3H, NCH_3_), 4.40 (s, 2H, CH_2_), 6.78–7.47 (m, 7H, ArH), 9.82 (br s, 1H, NHCO exchangeable with D_2_O). ^13^C NMR (DMSO) δ 11.4, 21.6, 27.8, 40.8, 56.8, 100.5, 106.8, 109.4, 125.8, 122.4 (2C), 127.6 (2C), 129.6, 135.6, 136.1, 147.8, 151.8, 168.4. ESI/MS *m*/*z* 323 [M + H]^+^, 345 [M + Na]^+^. Anal. Calcd. (C_19_H_22_N_4_O.HCl) C, H, N.

N-(4-(aminomethyl)benzyl)-N,1,2-trimethyl-1H-benzo[d]imidazol-5-amine (**31**)

This compound was prepared starting from **48** following the procedure described for **19**: an igroscopic solid was obtained (70% yield). The hydrochloride salt was transformed into oxalate salt, which was recrystallized from 2-PrOH, to obtain a white solid (m.p. 208–210 °C). ^1^H NMR (DMSO) δ 2.57 (s, 3H, CH_3_), 3.00 (s, 3H, CH_3_NCH_2_), 3.69 (s, 3H, NCH_3_), 3.98 (s, 2H, CH_2_NH_2_), 4.58 (s, 2H, CH_2_N), 6.79–7.40 (m, 7H, ArH), 8.22 (br s, 2H, NH_2_ exchangeable with D_2_O). ^13^C NMR (DMSO) δ 13.7, 29.9, 41.1, 45.9, 56.7, 100.6, 109.7, 110.8, 127.9, 128.3 (2C), 128.8 (2C), 130.8, 137.4, 141.6, 148.5, 151.4. ESI/MS *m*/*z* 281 [M + H]^+^. Anal. Calcd. (C_18_H_22_N_4_.2H_2_C_2_O_4_) C, H, N.

N-(3-(((1,2-dimethyl-1H-benzo[d]imidazol-5-yl)amino)methyl)benzyl)acetamide (**32**)

This compound was prepared starting from **55** following the procedure described for **22**: an oil was obtained (63% yield). The free base was transformed into the oxalate salt, which was recrystallized from 2-PrOH, to obtain a white solid (m.p. 198–200 °C). ^1^H NMR (CD_3_OD) δ 2.02 (s, 3H, CH_3_CO), 2.60 (s, 3H, CH_3_), 3.80 (s, 3H, NCH_3_), 4.43 (d, 2H, CH_2_N), 7.48–7.98 (m, 7H, ArH), 8.36 (br t, 1, NH exchangeable with D_2_O), 10.78 (br s, 1H, NHCO exchangeable with D_2_O). ^13^C NMR (CD_3_OD) δ 10.3, 21.1, 30.3, 42.0, 112.5, 112.8, 125.6, 126.7, 126.9, 127.9, 128.6, 130.2, 131.2, 135.8, 138.9, 142.9, 152.8, 165.3, 171.6. ESI/MS *m*/*z* 337 [M + H]^+^, 359 [M + Na]^+^, 676 [2M + H]^+^, 695 [2M + Na]^+^. Anal. Calcd. (C_19_H_20_N_4_O_2_.H_2_C_2_O_4_) C, H, N.

N-(3-(((1,2-dimethyl-1H-benzo[d]imidazol-5-yl)(methyl)amino)methyl)benzyl) acetamide (**33**)

This compound was prepared starting from **56** following the procedure described for **22**: an oil was obtained (62% yield). The free base was transformed into the hydrochloride salt, which was recrystallized from 2-PrOH, to obtain a white solid (m.p. 255–256 °C). ^1^H NMR (DMSO) δ 1.80 (s, 3H, CH_3_CO), 2.41 (s, 3H, CH_3_), 3.37 (s, 3H, CH_3_NCO), 3.61 (s, 3H, NCH_3_), 4.09 (d, 2H, CH_2_N), 6.99–7.38 (m, 7H, ArH), 8.20 (br t, 1, NH exchangeable with D_2_O). ^13^C NMR (DMSO) δ 12.1, 22.9, 31.5, 39.1, 42.1, 112.9, 113.4, 125.3, 126.9, 127.4, 128.2, 128.7, 130.7, 131.2, 136.5, 139.9, 142.7, 153.3, 169.6, 170.1. ESI/MS *m*/*z* 351 [M + H]^+^, 373 [M + Na]^+^, 723 [2M + Na]^+^. Anal. Calcd. (C_20_H_22_N_4_O_2_.HCl) C, H, N.

N-[3-(1,3-dioxolan-2-yl)benzyl]acetamide (**35**)

This compound was prepared starting from **34** following the procedure described for **22**: an oil was obtained (62% yield). ^1^H NMR (DMSO) δ 1.83 (s, 3H, CH_3_), 3.97 (m, 4H, CH_2_O), 4.28 (d, 2H, CH_2_N), 5.69 (s, 1H, OCHO), 7.18–7.83 (m, 4H, ArH), 8.37 (br t, 1H, NH exchangeable with D_2_O).

Tert-butyl(3-(((1,2-dimethyl-1H-benzo[d]imidazol-5-yl)amino)methyl)benzyl) carbamate (**39**)

This compound was prepared starting from **37** and **38** following the procedure described for **17**: an oil was obtained (56% yield). ^1^H NMR (CDCl_3_) δ 1.43 (s, 9H, C(CH_3_)_3_), 2.57 (s, 3H, CH_3_), 3.65 (s, 3H, NCH_3_), 3.92 (br s, 1H, NHAr exchangeable with D_2_O), 4.28–4.33 (m, 4H, CH_2_NH), 4.97 (br s, 1H, NHCO exchangeable with D_2_O), 6.62–7.34 (m, 7H, ArH).

Tert-butyl(3-(((1,2-dimethyl-1H-benzo[d]imidazol-5-yl)(methyl)amino)methyl)benzyl)carbamate (**40**)

This compound was prepared starting from **39** following the procedure described for **21**: an oil was obtained (63% yield). ^1^H NMR (CD_3_OD) δ 1.41 (s, 9H, C(CH_3_)_3_), 2.40 (s, 3H, CH_3_), 2.87 (s, 3H, CH_3_NCH_2_), 3.62 (s, 3H, NCH_3_), 4.16 (s, 2H, CH_2_N), 4.40 (s, 2H, CH_2_NH), 6.81–7.27 (m, 7H, ArH).

1,2-dimethyl-N-(3-nitrobenzyl)-1H-benzo[d]imidazol-5-amine (**45**)

This compound was prepared starting from **43** and **38** following the procedure described for **17**: an oil was obtained (70% yield). ^1^H NMR (DMSO) δ 2.40 (s, 3H, CH_3_), 3.62 (s, 3H, NCH_3_), 4.05 (br s, 1H, NH exchangeable with D_2_O), 4.63 (s, 2H, CH_2_), 6.78–8.08 (m, 7H, ArH).

Tert-butyl(4-(((1,2-dimethyl-1H-benzo[d]imidazol-5-yl)amino)methyl)benzylcarbamate (**46**)

This compound was prepared starting from **44** and **38** following the procedure described for **17**: an oil was obtained (59% yield). ^1^H NMR (DMSO) δ 1.40 (s, 9H, C(CH_3_)_3_), 2.40 (s, 3H, CH_3_), 3.59 (s, 3H, NCH_3_), 4.06 (m, 2H, CH_2_NH), 4.12 (m, 2H, CH_2_N), 5.38 (br s, 1H, NHCO), 5.82 (br t, 1H, NH exchangeable with D_2_O), 6.60–7.41 (m, 7H, ArH).

***N,1,2-trimethyl-N-(3-nitrobenzyl)-1H-benzo[d]imidazol-5-amine (47).***

This compound was prepared starting from **45** following the procedure described for **21**: an oil was obtained (83% yield). ^1^H NMR (DMSO) δ 2.40 (s, 3H, CH_3_), 3.00 (s, 3H, CH_3_NCH_2_), 3.62 (s, 3H, NCH_3_), 4.63 (s, 2H, CH_2_), 6.78–8.08 (m, 7H, ArH).

Tert-butyl(4-(((1,2-dimethyl-1H-benzo[d]imidazol-5-yl)(methyl)amino)methyl)benzyl) carbamate (**48**)

This compound was prepared starting from **46** following the procedure described for **21**: an oil was obtained (63% yield). ^1^H NMR (CD_3_OD) δ 1.41 (s, 9H, C(CH_3_)_3_), 2.42 (s, 3H, CH_3_), 2.93 (s, 3H, CH_3_NCH_2_), 3.60 (s, 3H, NCH_3_), 4.08 (s, 2H, CH_2_N), 4.43 (s, 2H, CH_2_NH), 4.20 (br s, 1H, NHCO), 6.82–7.38 (m, 7H, ArH).

N-(1,2-dimethyl-1H-benzimidazol-5-yl)-4-nitrobenzamide (**51**)

A solution of **38** (7.18 mmol) in DMF (5 mL) was added to a solution of 4-nitrobenzoic acid **49** (5.98 mmol), HOBt (7.18 mmol), EDCI7#xB7;HCl (7.18 mmol) and N-methylmorpholine (1.31 mL, 11.96 mmol) in DMF (10 mL). The reaction mixture was stirred at r.t. for 22 h. The solvent was evaporated and the residue was treated with 2N NaOH (20 mL), extracted with dichloromethane (3 × 20 mL), washed with brine, dried over Na_2_SO_4_, and concentrated to get a residue, which was purified by flash chromatography eluting with chloroform/MeOH (95:5) to obtain an oil (60% yield). ^1^H NMR (DMSO) δ 2.41 (s, 3H, CH_3_), 3.62 (s, 3H, NCH_3_), 6.95–7.37 (m, 7H, ArH), 7.98 (br s, 1H, NH exchangeable with D_2_O).

3-Cyano-N-(1,2-dimethyl-1H-benzimidazol-5-yl)benzamide (**52**) 

This compound was prepared starting from **50** and **38** following the procedure described for **51**: an oil was obtained (67% yield). ^1^H NMR (DMSO) δ 2.40 (s, 3H, CH_3_), 3.67 (s, 3H, NCH_3_), 7.40–8.20 (m, 7H, ArH), 10.19 (br s, 1, NH exchangeable with D_2_O).

1,2-Dimethyl-N-(4-nitrobenzyl)-1H-benzo[d]imidazol-5-amine (**53**)

Borane dimethyl sulfide complex (1.5 mL, 16.10 mmol) was added to a suspension of **51** (3.22 mmol) in THF (30 mL). The reaction mixture was heated to 80 °C for 3h. The reaction was quenched with dropwise addition of 2N HCl (10 mL) and then basified with 2N NaOH (25 mL). The compound was extracted with AcOEt (3 × 20 mL), washed with water, and dried over Na_2_SO_4_. Evaporation of the solvent yielded a residue, which was purified by flash chromatography eluting with chloroform/MeOH (95:5) to get oil (39% yield). ^1^H NMR (DMSO) δ 2.40 (s, 3H, CH_3_), 3.60 (s, 3H, NCH_3_), 4.40 (s, 2H, CH_2_), 6.03 (br s, 1H, NH exchangeable with D_2_O), 6.52–8.20 (m, 7H, ArH).

3-Cyano-N-(1,2-dimethyl-1H-benzo[d]imidazol-5-yl)-N-methylbenzamide (**54**)

Then 60% dispersion of NaH in mineral oil (9.66 mmol) was added in portions to a solution of **52** (4.83 mmol) in DMF (20 mL) at 0 °C. After 30 min under stirring, a solution of CH_3_I (0.37 mL, 5.79 mmol) in DMF (5 mL) was added to the suspension. The reaction mixture was stirred for 3 h at room temperature, then was quenched with brine and extracted with AcOEt (3 × 20 mL). The organic layer was washed with brine, dried on Na_2_SO_4_, concentrated under vacuo, and purified by flash chromatography using chloroform/MeOH (95:5) as an eluent, to give an oil (69% yield). ^1^H NMR (DMSO) δ 2.42 (s, 3H, CH_3_), 3.38 (s, 3H, CH_3_NCO), 3.62 (s, 3H, NCH_3_), 7.02–7.77 (m, 7H, ArH).

3-(Aminomethyl)-N-(1,2-dimethyl-1H-benzo[d]imidazol-5-yl)benzamide (**55)**

This compound was prepared starting from **52** following the procedure described for **20**: an oil was obtained (85% yield). ^1^H NMR (CD_3_OD) δ 2.43 (s, 3H, CH_3_), 3.61 (s, 3H, NCH_3_), 3.82 (s, 2H, CH_2_N), 7.22–7.98 (m, 7H, ArH), 8.72 (br s, 2H, NH_2_ exchangeable with D_2_O), 10.90 (br s, 1H, NHCO exchangeable with D_2_O).

3-(Aminomethyl)-N-(1,2-dimethyl-1H-benzo[d]imidazol-5-yl)-N-methylbenzamide (**56**)

This compound was prepared starting from **54** following the procedure described for **20**: an oil was obtained (81% yield). ^1^H NMR (CD_3_OD) δ 2.56 (s, 3H, CH_3_), 3.50 (s, 3H, NCH_3_), 3.60 (br s, 2H, NH_2_ exchangeable with D_2_O), 3.68 (s, 3H, CH_3_NCO), 4.82 (s, 2H, CH_2_N), 7.04–7.37 (m, 7H, ArH).

### 3.2. NAPRT Activity Screening Assay

NAPRT activity was assayed by using a continuous coupled fluorometric assay or a HPLC-based assay. The fluorometric assay was used for the initial screening of the compounds and its optimization and validation is described in our recent paper [35]. Briefly, it relies on an ancillary enzymatic system that converts the reaction product NAMN to NADH. In detail, NAMN is stoichiometrically adenylated to NAAD by the bacterial enzyme nicotinate mononucleotide adenylyltransferase (NadD), NAAD is amidated to NAD^+^ by NAD^+^ synthase (NadE) and finally, NAD^+^ is reduced to NADH by alcohol dehydrogenase (ADH). The assay mixture (0.2 mL) contained 100 mM HEPES/NaOH buffer, pH 7.5, 10 mM MgCl_2_, 0.5 mg/mL bovine serum albumin, 75 mM ethanol, 30 mM semicarbazide, 4.5 mM NH_4_Cl, 0.5 mM compound in DMSO (ensuring 2% final concentration of DMSO in the reaction mixture), 0.4 U/mL B. anthracis NadD, 0.2 U/mL B. anthracis NadE, 12.5 U/mL yeastADH, 1 mM ATP, 0.4 mM PRPP, 0. 2 mM NA and 0.6 × 10^−3^ U/mL NAPRT. Human recombinant NAPRT was prepared as described in [27]. One Unit (U) of NAPRT is the amount of enzyme that catalyzes the formation of 1 µmol NAMN per minute at 37 °C. The ancillary enzymes NadD and NadE were prepared in the form of recombinant proteins as described in [36]. NADH fluorescence was monitored continuously at 37 °C for 30 min, using a 96-well plate in a microplate reader at excitation and emission wavelengths of 340 nm and 460 nm, respectively. Compounds **17–33** were found to interfere at the assay’s wavelengths and therefore their effect on NAPRT was tested by using an HPLC-based assay, which allows the direct measurement the product of the NAPRT-catalyzed reaction, i.e., NAMN. To this end, reaction mixtures containing 50 mM HEPES/NaOH, pH 7.5, 10 mM MgCl_2_, 0.5 mg/mL BSA, 1 mM ATP, 0.2 mM NA, 0.4 mM PRPP, 1 mM compound in DMSO (2% DMSO in the reaction mixture) and 1.7 × 10^−3^ U/mL NAPRT were incubated at 37 °C. At different incubation times, the reaction was stopped by acidification with 0.6 M HClO_4_. After 10 min on ice, samples were centrifuged (13,000× *g*, 2 min) and the supernatants were neutralized with 0.8M K_2_CO_3_ and centrifuged again to remove precipitated KClO_4_. Samples were injected onto a Supelcosil LC-18-S column (5 µm; 250 × 4.6 mm) equilibrated with 100 mM potassium phosphate, pH 6.0, containing 8 mM tetrabutylammonium hydrogen sulfate (buffer A) and eluted at flow-rate of 1 mL/min under the following gradient conditions: 3 min at 100% buffer A; 1 min up to 90% buffer B (buffer A plus 30% methanol); 10 min hold at 90% buffer B, 1 min up to 100% buffer A, followed by re-equilibration with buffer A for 7 min. The column temperature was maintained at 8 °C, and eluate absorbance was monitored at 260 nm. The enzyme activity was calculated by referring to a NAMN standard.

Both assays were optimized to ensure conditions of the initial velocity. In both assays, reaction mixtures included positive controls (NAPRT and no compound), negative controls (no NAPRT and no compound), and compound controls (compound and no NAPRT). The coupled fluorometric assay also included a control to test the effect of the compound on the ancillary system. The percentage inhibition and the fold activation were calculated relative to the controls.

### 3.3. Kinetic Analyses

Kinetic analyses were performed by using the HPLC assay. An appropriate enzyme amount was used in order to provide a substrate consumption below 10% of the initial concentration after 10 min incubation at any substrate concentration used. In addition, withdrawals from the assay mixture at two different incubation time was always performed to ensure a linear time frame. Kinetic parameters were calculated from the initial velocity data by using the Lineweaver–Burk plot [37].

### 3.4. In Silico Studies

In silico studies were performed using ad hoc tools implemented in Molsoft ICM-Pro version 3.9, a complete molecular modeling and docking software package [38]. The crystal structure of human dimeric NAPRT was used as a template (PDB: 4yub). The structure was prepared with standard procedures, including removing water molecules, adding hydrogens, and optimizing orientation and protonation states. Initially, the substrate binding site was defined using the ICM pocket finder tool with a relaxed tolerance. This allowed us to generate a 30 Å grid encompassing the enzyme active site and adjacent regions. The ligand and a few receptor residues (i.e., Arg171^A^ and Arg172^A^) were set as fully flexible. A 3D pharmacophore model of docked modulators was generated using the Atomic Property Fields (APF) superposition/alignment method [39].

## 4. Conclusions

In the present study, more than 200 small molecules available in our labs and characterized by different chemical scaffolds were screened to discover new NAPRT modulators. Few weak NAPTR inhibitors have been identified, including the 1,2-dimethylbenzimidazole compound **17**. From a comprehensive SAR study performed on compound **17**, the 1,2-dimethylbenzimidazole nucleus proved to be a versatile scaffold to obtain NAPRT inhibitors or activators, depending on the substituents inserted in position 5. Indeed, derivatives **24**, **31**, and **32** emerged as NAPRT activators, while **18** showed the highest % inhibition within the series and behaved as a noncompetitive inhibitor towards NA and a mixed inhibitor towards PRPP. Docking studies of the best inhibitors **17** and **18** were performed on the NAPRT structure to get insights into the ligand-pocket interactions. Moreover, a preliminary pharmacophore model was built to shed light on the shift from inhibitors to activators and guide their future optimization. From in vitro pharmacokinetic studies, compound **18** showed an overall good ADME profile, displaying high kinetic solubility at pH 7.4, low protein binding in both human and mouse plasma, and high metabolic stability in human liver cell microsomes. However, despite its good MDCKII cell permeability, compound **18** is a P-gp substrate, and this might limit tumor cell absorption. Therefore, the need for effective NAPRT inhibitors makes this compound a useful starting point to obtain new derivatives with improved NAPRT inhibitory potency and reduced P-gp affinity, potentially useful as antitumor agents. Moreover, being **24**, **31**, and **32** the first NAPRT activators identified so far, they might represent promising starting points whose optimization might lead to pharmacological tools to explore the therapeutical potential of the enzyme’s pharmacological activation.

## Figures and Tables

**Table 1:** NAPRT inhibition/activation effect of compounds **17–33** ^a^.

Compound	Structure	% Inhibition	-Fold Stimulation
**17**	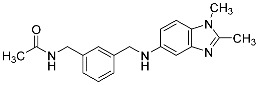	30.3 ± 6.0	-^b^
**18**	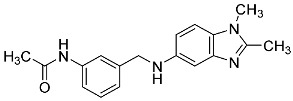	46.1 ± 8.1	-
**19**	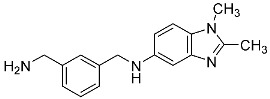	-	-
**20**	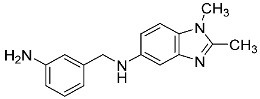	-	1.25 ± 0.05
**21**	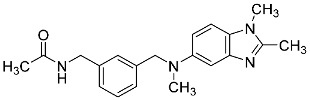	-	-
**22**	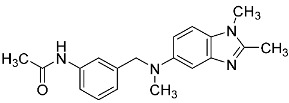	-	-
**23**	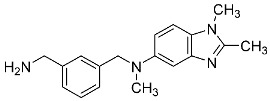	-	1.14 ± 0.05
**24**	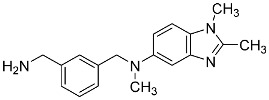	-	1.44 ± 0.04
**25**	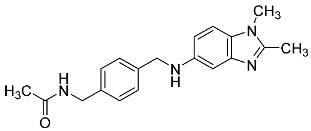	13.0 ± 3.1	-
**26**	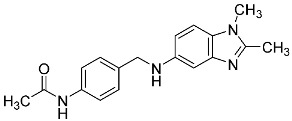	-	-
**27**	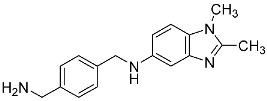	-	-
**28**	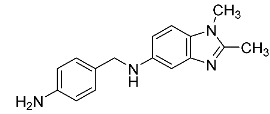	-	-
**29**	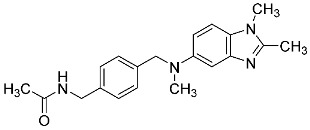	12.0 ± 2.2	-
**30**	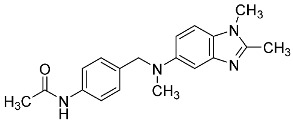	-	-
**31**	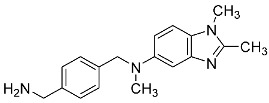	-	1.58 ± 0.07
**32**	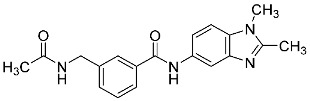	-	1.47 ± 0.07
**33**	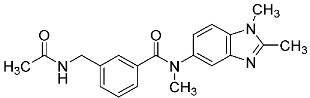	-	-

^a^ NAPRT activity was assayed as described in Materials and Methods. ^b^ Not active, it is referred to compounds showing less than 10% inhibition or less than 1.10-fold stimulation (values are reported in Table S1).

**Table 2:** ADME parameters of compound **18**.

	18	
Kinetic Solubility (pH 7.4)	µM	312
	mg/mL	0.1302
Protein Binding ^a^	Human plasma (FU) ^b^	14.6
	Mouse plasma (FU) ^b^	>50
Hepatic Intrinsic Clearance (CLi) in Liver Microsomes	Human Cli (µL/min/mg protein)	<9.92
	WSM ^c^ in vivo prediction (mL/min/kg)	6.83
	Mouse Cli (µL/min/mg protein)	120
	WSM ^c^ in vivo prediction (mL/min/kg)	26.9
Permeability ^d^	Papp A-B MDCKII (nm/sec)	38.3
	Papp B-A MDCKII (nm/sec)	65.9
	Efflux Ratio MDCKII (B-A/A-B)	1.7
	Papp A-B MDCKII-MDR1 (nm/sec)	3.39
	Papp B-A MDCKII-MDR1 (nm/sec)	174
	Efflux Ratio MDCKII-MDR1 (B-A/A-B)	51.3

^a^ Experiment performed at 0.5 μM; ^b^ FU = fraction unbound; ^c^ WSM = well-stirred model; ^d^ Papp = passive membrane permeability, A-B = apical to basolateral, B-A = basolateral to apical.

## Data Availability

Data is contained within the article and Supplementary Materials.

## Supplementary Materials

The following supporting information can be downloaded at: https://www.mdpi.com/article/10.3390/ph16020189/s1, Table S1: NAPRT inhibition/activation effect of tested compounds; Table S2: Elemental analysis results for compounds **17**−**33**; materials and general methods for the synthesis and chemical characterization of the final compounds and intermediates.
