# Supplementary material for: Properly Substituted Benzimidazoles as a New Promising Class of Nicotinate Phosphoribosyltransferase (NAPRT) Modulators
Source: Pharmaceuticals (Basel). 2023 Jan 27;16(2):189. doi: 10.3390/ph16020189 (PMC9967085; doi:10.3390/ph16020189)
Supplement: Supplementary file 1 [file pharmaceuticals-16-00189-s001.zip › pharmaceuticals-2144882-supplementary.pdf]

SUPPLEMENTARY MATERIALS

Properly Substituted Benzimidazoles as a New Promising Class of Nicotinate Phosphoribosyltransferase (NAPRT) Modulators

Cecilia Baldassarri,[1] Gianfabio Giorgioni,[1] Alessandro Piergentili,[1] Wilma Quaglia,[1] Stefano Fontana,[2] Valerio Mammoli,[2] Gabriele Minazzato,[3] Elisa Marangoni,[3] Massimiliano Gasparrini,[3] Leonardo Sorci,[3] Nadia Raffaelli,*[3] Loredana Cappellacci,[1] Riccardo Petrelli,*[1] Fabio Del Bello[1]

[1]School of Pharmacy, Chemistry Interdisciplinary Project (ChIP), University of Camerino, via Madonna delle Carceri, 62032 Camerino, Italy

[2]Center for Drug Discovery and Development-DMPK, Aptuit, an Evotec Company, via A. Fleming 4, 37135 Verona, Italy

[3]Department of Agriculture, Food and Environmental Sciences, Polytechnic University of Marche, via Brecce Bianche 10, Ancona, Italy

Table of Contents:

Table S1: NAPRT inhibition/activation effect of tested compounds……………………………S2

Table S2: Elemental analysis results for compounds **17-33**..………..…………………..…..……S4

Materials and general methods for the synthesis and chemical characterization of the final compounds and interemediates……………………………………………………………...S5

Table S1. NAPRT inhibition/activation effect of tested compounds

Compound	Structure	% Inhibition	-Fold stimulation
**17**	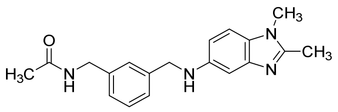	30.3 ± 6.0	-
**18**	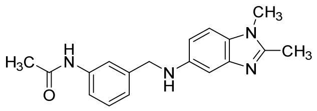	46.1 ± 8.1	-
**19**	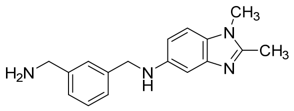	0.6 ± 0.9	-
**20**	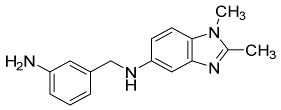	-	1.25 ± 0.05
**21**	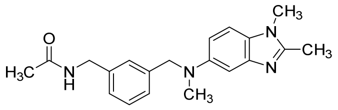	0	0
**22**	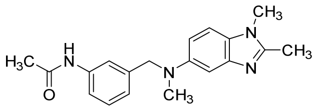	5.0 ± 1.2	-
**23**	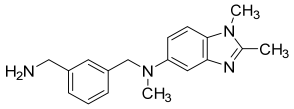	-	1.14 ± 0.05
**24**	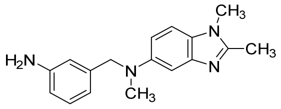	-	1.44 ± 0.04
**25**	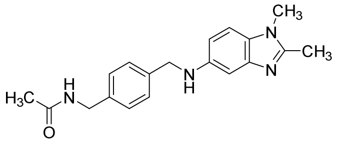	13.0 ± 3.1	-
**26**	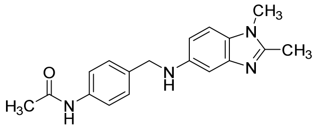	1.3 ± 0	-
**27**	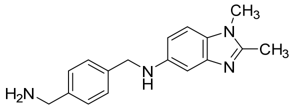	1.9 ± 0.9	-

**28**	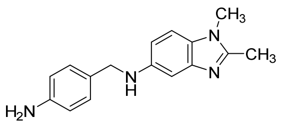	0	0
**29**	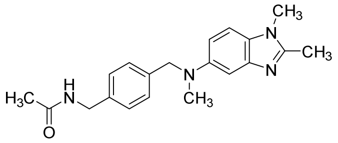	12.0 ± 2.2	-
**30**	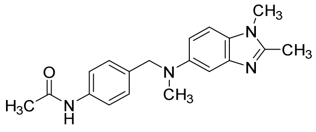	0	0
**31**	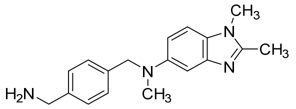	-	1.58 ± 0.07
**32**	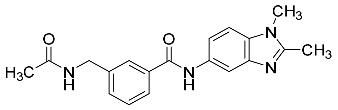	-	1.47 ± 0.07
**33**	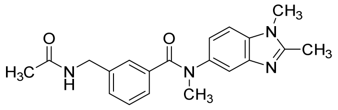	-	1.08 ± 0.03

Table S2. Elemental analysis results for compounds **17-33**.

Compd	Formula	Calcd			Found		
		C%	H%	N%	C%	H%	N%
**17**	$C_{19}H_{22}N_4O.2HCl$	57.73	6.12	14.17	57.99	5.98	13.90
**18**	$C_{18}H_{20}N_4O.2HCl$	56.70	5.82	14.69	56.42	5.99	14.39
**19**	$C_{17}H_{20}N_4.2HCl$	57.80	6.28	15.86	57.65	6.13	15.91
**20**	$C_{16}H_{18}N_4.2H_2C_2O_4$	53.81	4.97	12.55	54.03	4.77	12.68
**21**	$C_{20}H_{24}N_4O.H_2C_2O_4$	61.96	6.15	13.14	62.15	6.00	13.37
**22**	$C_{19}H_{22}N_4O.2HCl$	57.73	6.12	14.17	58.01	6.23	13.94
**23**	$C_{18}H_{22}N_4.2H_2C_2O_4$	55.69	5.52	11.81	55.91	5.70	12.02
**24**	$C_{17}H_{20}N_4.H_2C_2O_4$	61.61	5.99	15.13	61.84	6.11	15.33
**25**	$C_{19}H_{22}N_4O.2HCl$	57.73	6.12	14.17	57.96	6.21	14.02
**26**	$C_{18}H_{20}N_4O.2H_2C_2O_4$	54.10	4.95	11.47	54.23	5.02	11.25
**27**	$C_{17}H_{20}N_4.2H_2C_2O_4$	54.78	5.25	12.17	54.90	5.16	12.33
**28**	$C_{16}H_{18}N_4$	72.15	6.81	21.04	72.39	6.95	20.82
**29**	$C_{20}H_{24}N_4O.2HCl$	58.68	6.40	13.69	58.38	6.23	13.60
**30**	$C_{19}H_{22}N_4O.HCl$	63.59	6.46	15.61	63.76	6.34	15.84
**31**	$C_{18}H_{22}N_4.2H_2C_2O_4$	57.73	6.12	14.17	57.79	6.01	14.01
**32**	$C_{19}H_{20}N_4O_2.H_2C_2O_4$	59.15	5.20	13.14	59.44	5.30	13.03
**33**	$C_{20}H_{22}N_4O_2.HCl$	62.09	5.99	14.48	62.35	5.87	14.69

Materials and general methods for the synthesis and chemical characterization of the final compounds and intermediates.

All the reagents have been purchased and used without further purification. Melting points were taken in glass capillary tubes on a Büchi SMP-20 apparatus. Elemental analyses (C, H, N, and S) were recorded on FLASH 2000 instrument (ThermoFisher Scientific). The elemental composition of the compounds agreed to within ±0.4% of the calculated value. ^{1}H-NMR spectra were recorded either with a Bruker 500 Ascend (Bruker BioSpin Corporation, Billerica, MA, USA) and Varian Mercury AS400 instruments, and chemical shifts (ppm) are reported relative to tetramethylsilane. Spin multiplicities are given as s (singlet), d (doublet), dd (double doublet), t (triplet), or m (multiplet). IR spectra were recorded on PerkinElmer 297 instrument and spectral data (not shown because of the lack of unusual features) were obtained for all compounds reported and are consistent with the assigned structures. ESI-MS spectra were recorded in the positive- [ESI-MS(+)] or negative-ion [ESIMS(−)] mode on a Waters Micromass ZQ spectrometer equipped with a single quadrupole (Waters Corporation, Milford, MA, USA), using a methanol mobile phase. The compounds were added to reagent grade methanol to give approximately 0.1 mM solutions. These solutions were injected (1 μL) into the spectrometer fitted with an autosampler. The pump delivered the solutions to the mass spectrometer source at a flow rate of 200 μL/min, and nitrogen was employed both as a drying gas and as a nebulizing gas. Capillary voltage was typically 2500 V. The temperature of the source was 100 °C, while the temperature of the desolvation was 400 °C. All reactions were monitored by thin-layer chromatography using silica gel plates (60 F254; Merck), visualizing with ultraviolet light. Chromatographic separations were performed on silica gel columns (Kieselgel 40, 0.040−0.063 mm, Merck) by flash chromatography. Compounds were named following IUPAC rules as applied by ChemBioDraw Ultra (version 12.0) software for systematically naming organic chemicals. The purity of the novel compounds was determined by combustion analysis and was ≥95%.
